# 

**DOI:** 10.3325/cmj.2012.53.643

**Published:** 2012-12

**Authors:** Vladimir Trkulja, Siniša Car

**In Reply:** We understand that the *Croatian Medical Journal* has received a Letter to the Editor with comments on our recently published study (1) and we thank you for the opportunity to immediately respond to these comments.

We thank Drs. Zhou, Wu, and Fang for their interest in our work, but we fail to see that their comments contain any relevant point that would in any way change/question the methodology and conclusions of our work.

Zhou and colleagues end their first paragraph stating that the “…conclusion should be interpreted with caution” (presumably having in mind that the results of our work should be interpreted with caution). We agree, and we did exercise a considerable caution. First, considering that multivariate models in different studies included different independent variables (besides uric acid, UA), we concluded that for a more precise/accurate estimate of the strength of the independent UA-acute myocardial infarction (AMI)-outcome association further studies were needed. We also concluded that further studies were needed in different settings, ie, STEMI, NSTEMI, mode of treatment etc. Second, we pointed-out that, particularly regarding the long-term outcomes (survival), on-admission serum UA might not be of such an interest as long-term development of the UA values during the post-AMI period. Unfortunately, no single study so far has addressed serum UA as a time-varying factor (covariate). On the other hand, we believe that the fact that 9 separate studies (including all combinations of the AMI type and treatment procedures and short-term, medium-term and long-term outcomes) all found an independent association of UA and adverse AMI outcomes – speaks for itself. Indeed, 9 studies with around 8000 patients might be an insufficient number for a definite, robust generalization about the UA-AMI outcome relationship (and their individual limitations were adequately considered), but the results of the individual studies as well as of the pooled analysis are more than indicative.

At the beginning of the second paragraph, Zhou and colleagues refer to 3 or 4 studies referring to the association between serum UA and coronary heart disease (CHD) and conclude that literature data are inconsistent regarding the question whether serum UA “predicts CHD.” In this respect, we would like to point out the following: a) our aim WAS NOT to assess whether serum UA was a predictor of occurrence of CHD (any clinical manifestation), since this issue had been thoroughly addressed, rather, we addressed another question: do on-admission (within 48 hours since the symptom onset) serum UA levels predict the outcome in patients affected by AMI? b) As for the relationship between serum UA and CHD (incidence, mortality), we draw the attention to the meta-analysis published in 2010 (2), cited also in our article It embraced 13 large prospective studies (with close to 300 000 participants) all of which, through multivariate models, assessed an independent association between UA and CHD. Based on 13 studies (pooled), higher UA was associated with a higher risk of CHD occurrence (any clinical form) and based on 9 studies (pooled), higher UA was associated with higher CHD-related mortality.

Next, Zhou and colleagues mention the possible beneficial (anti-oxidant) properties of UA. This is a well known fact and is addressed also in our work. Still, the fact remains that PROLONGED HIGH(ER) UA levels clearly are adversely related to many different cardiovascular diseases (regardless of the possible mechanistic explanation or lack of it). Further, Zhou and colleagues refer to one study that investigated the relationship between serum UA and the outcomes in acute ischemic stroke (IS) patients. In this respect, we would like to point out: a) acute IS and acute MI, although with many common underlying features are two DIFFERENT diseases (eg, there are differences in the relevance of individual known risk factors, secondary prevention treatments etc); we addressed only MI and made no implications regarding IS; b) there are numerous studies investigating UA and IS. One meta-analysis of 16 studies with around 250 000 subjects found UA to be independently associated with a higher incidence and higher IS-related mortality (3). A number of subsequent studies found UA to be associated with either better or worse outcomes, or found no association between UA and outcomes in IS-affected patients. But this has nothing to do with the questions related to UA as a predictor of outcomes in acute MI patients.

Finally, Zhou and colleagues warn about the “…limitations of meta-analysis…” due “...to non-random choice of individual studies”. We do not understand this comment at all: systematic review and meta-analysis IS ABOUT inclusion of ALL available studies (which we accomplished through a very thorough literature search) and their critical evaluation and, eventually (if feasible and justified), pooled analysis with a goal of obtaining the most realistic estimate (of an effect).

To conclude, the relationship between serum UA levels and various aspects of cardio- and cerebrovascular diseases has been extensively investigated. We addressed only one simple question: Do serum UA levels taken on-admission in patients affected by acute myocardial infarction predict the outcome? The available data and the critical assessment and pooled analysis that we performed strongly suggest that serum UA should be considered as an independent predictor regarding the short-term, medium-term, and long-term outcomes in these patients.
